# Trends and In‐Hospital Outcomes of Ventricular Tachycardia in Ischemic Versus Non‐Ischemic Dilated Cardiomyopathy—An Insight From the Nationwide Readmissions Database (2016–2020)

**DOI:** 10.1111/anec.70232

**Published:** 2026-08-31

**Authors:** Medhat Chowdhury, Sanchit Duhan, Rasheed Bahar, Mohamad Hasan Jawadi, Bijeta Keisham, Harini Lakshman, Marc Zughaib, Yasar Sattar, Christopher Bradley, Marcel Zughaib

**Affiliations:** ^1^ Department of Cardiology Henry Ford Providence Hospital Southfield Michigan USA; ^2^ Department of Cardiology University of Illinois Urbana‐Champaign, Carle Foundation Hospital Illinois USA; ^3^ Department of Internal Medicine Wayne State University Detroit Michigan USA; ^4^ Department of Medicine Conway Regional Medical Center Arkansas USA; ^5^ Department of Cardiology West Virginia University Morgantown West Virginia USA

**Keywords:** cardiogenic shock, cardiomyopathy, implantable cardiac defibrillator, ventricular tachycardia

## Abstract

**Background:**

Ventricular tachycardia (VT) is a major cause of morbidity and mortality in patients with structural heart disease, yet real‐world comparisons between ischemic cardiomyopathy (ICM) and non‐ischemic dilated cardiomyopathy (NIDCM) remain limited.

**Methods:**

The national readmission database (2016–2020) was used to identify hospitalizations for VT. Cohorts were stratified based on underlying cardiomyopathy. A Propensity Score Matching (PSM) model matched ICM to NIDCM patients. Pearson's x2 test was applied to PSM‐matched cohorts to compare outcomes.

**Results:**

We identified 622,092 VT hospitalizations, of which 575,312 (92.4%) had ICM and 46,780 (7.5%) had NIDCM. Outcomes were compared using multivariable logistic regression and propensity score matching (25,919 matched pairs). Despite being younger, NIDCM patients had higher in‐hospital mortality (10.4% vs. 9.6%, *p* < 0.001; aOR 1.08), stroke (1.7% vs. 1.2%, aOR1.42), acute heart failure (65% vs. 59%, aOR 1.36), acute kidney injury (46.1% vs. 41.7%, aOR1.35), endotracheal intubation (13.1% vs. 14.5%, aOR 1.06), ICD implantation (3.9% vs. 3.5%, aOR1.23), and VT ablation (1.0% vs. 0.5%, aOR1.76). Readmission rates were significantly higher in NIDCM at 30 days (10.4% vs. 8.7%, *p* = 0.019), 90 days (22.7% vs. 20.0%, *p* = 0.483), and 180 days (30.7% vs. 27.3%, *p* = 0.001). In contrast, ICM patients had higher rates of sudden cardiac arrest (10.6% vs. 5.9%, aOR0.55), major adverse cardiovascular events (78.9% vs. 52.2%, aOR0.24), cardiogenic shock (18.4% vs. 12.1%, aOR0.59), ECMO (0.9% vs. 0.4%, aOR0.48), and LVAD use (0.5% vs. 0.3%, aOR0.58). Mortality declined over time in ICM (8.5% in 2016 to 8.2% in 2020, *p*‐trend = 0.006), but not in NIDCM.

**Conclusion:**

These findings highlight distinct clinical profiles and emphasize the need for targeted management strategies for VT based on the cardiomyopathy subtype.

## Graphical abstract

Lower rates of sudden cardiac arrest and major adverse cardiovascular events in NIDCM patients. Higher rates of in‐hospital mortality, stroke, and heart failure‐related admissions in NIDCM patients.

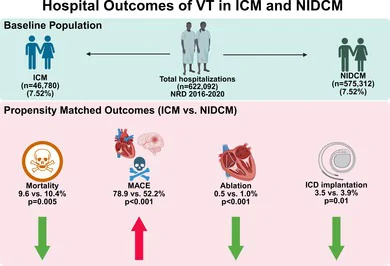

## 1 Introduction

Ventricular tachycardia (VT) remains a major cause of morbidity and mortality among patients with structural heart disease, particularly those with ischemic cardiomyopathy (ICM) and non‐ischemic dilated cardiomyopathy (NIDCM) (Zipes and Wellens 1998). In recent years, there have been significant advancements in VT management, including refined catheter ablation strategies, endo‐epicardial approaches, high‐resolution mapping, and improved implantable cardioverter defibrillator (ICD) risk stratification (Muser et al. 2016; Proietti et al. 2021; Di Marco et al. 2021). In parallel, earlier and complete coronary revascularization in ICM has been associated with a reduced arrhythmic burden and sudden cardiac death risk (Thomsen et al. 2023).

Despite these advances, contemporary real‐world data comparing procedural utilization, mortality, and readmission outcomes in VT patients with ICM versus NIDCM are lacking. In this study, we leveraged the Nationwide Readmission Database (NRD) from 2016 to 2020 to evaluate trends in in‐hospital outcomes, resource use, and arrhythmia‐related interventions across these two cardiomyopathy subtypes during a period of rapid therapeutic evolution.

## 2 Methods

### 2.1 Study Design and Population

The Nationwide Readmission Database (NRD) from 2016 to 2020 was utilized for this study. NRD is maintained by the Agency for Healthcare Research and Quality (AHRQ) and provides data on roughly 35 million weighted hospitalizations. It is a nationally representative administrative database of the United States comprising discharge and readmission records of 62.2% of all hospitalizations. International Classification of Disease, Tenth Edition, Clinical Modification (ICD‐10‐CM) was used to select patients admitted with ventricular tachycardia (ICD‐10 code I47.0, which denotes “Re‐entry ventricular arrhythmia,” and I47.2, which corresponds to “Ventricular tachycardia, unspecified”). These codes were adapted from previous readmission database studies (Moustafa et al. 2023; Tan et al. 2024). ICD‐10 Coding System (ICD‐10‐PCS) codes were used to identify ICM (I25.5) and DCM (I42.0). The NIDCM cohort was defined by excluding all patients in the dilated cardiomyopathy group with a documented history of obstructive coronary artery disease (CAD), prior percutaneous coronary intervention (PCI), or coronary artery bypass grafting (CABG). ICD‐10 codes used to identify the study population and the primary and secondary outcomes are included in Table S1. Trend analyses of in‐hospital mortality, resource utilization, and interventions during index hospitalizations were obtained from the entire study population. However, for our outcomes analysis, cohorts were created based on the types of cardiomyopathy, such as ICM and NIDCM. No chart‐level validation or sensitivity analysis restricted to I47.2 alone was performed because the NRD is an administrative database without access to clinical records or electrocardiographic confirmation. Therefore, the specificity of the combined I47.0/I47.2 coding definition for VT could not be independently confirmed.

**FIGURE 1:**
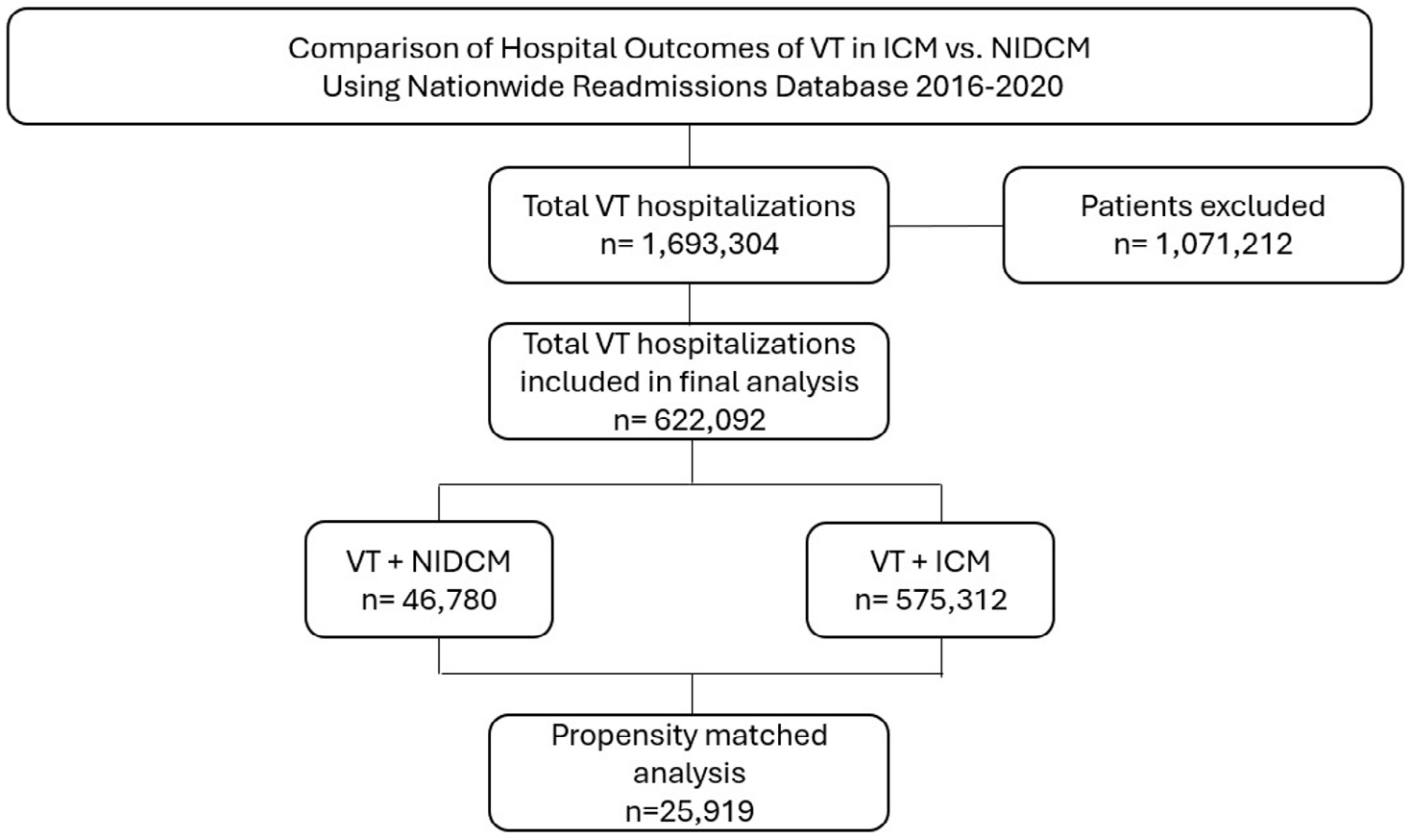
Study characteristics.

Furthermore, all duplicates and minors (< 18 years) were identified and excluded. Subsequently, we classified the patient population into two groups: VT patients with ICM and VT patients with NIDCM (Figure 1). Individual cases were identified using the unique identifier code. The number of days to intervention/procedure and length of stay (LOS) variables were used to calculate the readmission day of the same patient population. Data were used in their totality for analysis at index admission. As NRD is annualized and only patients admitted within the same calendar year can be identified, we sequentially included the first 11‐, 9‐, and 6‐month data from each year to ensure all patients have 30‐, 90‐, and 180‐day follow‐ups, respectively. Observations with a count < 11 were not reported per HCUP reporting guidelines.

### 2.2 Baseline Characteristics

We identified adult patients (age ≥ 18 years) who were admitted between 2016 and 2020 with VT. Baseline patient characteristics (i.e., age, sex, and patient comorbidities) were analyzed. Hospital characteristics analyzed included bed size, teaching status, and urban–rural designation.

### 2.3 Study Outcomes

The primary outcome was the difference in in‐hospital mortality between VT patients with ICM and NIDCM. Secondary outcomes included other complications during the index hospitalization: sudden cardiac arrest (SCA), acute stroke, MACE, ICD implantation, ablation, cardiogenic shock, acute CHF, left ventricular assist device (LVAD) utilization, extracorporeal membrane oxygenation (ECMO) utilization, cardiac transplantation, AKI, and endotracheal intubation. Length of stay, propensity‐matched 30‐ and 180‐day readmission rates, trends of VT‐related mortality, ICD implantation, and ablation for VT in ICM and NIDCM. The definitions of study outcomes are provided in Table S2.

### 2.4 Statistical Analysis

Descriptive statistics were used to summarize continuous and categorical variables. Categorical variables were expressed as percentages and frequencies and compared using Pearson's chi‐square (x^2^) test (Pearson 1900). After assessing the distribution of data with histogram analysis (Figure S1), continuous variables were compared using the independent sample *t*‐test analysis (for normally distributed) or the Mann–Whitney U test (Wilcoxon rank sum test) for non‐parametric distribution (Mann and Whitney 1947).

Patient demographics, comorbidities, and study outcomes were compared between ICM and NIDCM cohorts. The frequency of missing values was summarized, and Little's test for missing completely at random (MCAR) was used to screen for missing‐data patterns. A non‐significant *p*‐value (*p* > 0.05) represented randomly missing, while a significant *p*‐value (*p* < 0.05) indicated missing not at random (MNAR) (Little 1988).

After handling missing data, unadjusted and adjusted odds ratios were analyzed for in‐hospital outcomes using univariate and multivariate logistic regression for study cohorts. We measured the adjusted odds ratios for in‐hospital outcomes with *p*‐values < 0.05 and confidence intervals not crossing 1 by matching comorbidities and demographics to limit confounding. We used univariate screening to build the regression model; *p*‐values < 0.2 were used as the cutoff for including covariates in the final multivariate regression model (Mickey and Greenland 1989). The multicollinearity among independent variables was assessed by measuring the variance inflation factor (VIF) and tolerance (1/VIF). VIF > 5 and a tolerance value < 0.2 were taken as indicators of significant correlation among independent variables (O'brien 2007). Covariates included in multivariate regression are listed in Table S4.

To validate our regression findings, we performed a Propensity Score Matching (PSM) model, implementing complete Mahalanobis Distance Matching within the Propensity Score Caliper (0.2) (Rubin 2001), matched VT patients with underlying ICM and VT with NIDCM. The variables utilized for the multivariate regression model were included in the PSM model to obtain matched cohorts. This step was taken to cross‐verify our multivariate regression outcomes with propensity‐matched results. Pearson's x^2^ test was applied to the matched cohorts to compare outcomes. Teffects psmatch was used to create a graphical box plot showing the balance of matching variables across both cohorts (Figure S2). The matching variables (demographics, disease severity, mortality risk, and 15 different baseline comorbidities) used in the PSM‐2 module are listed in Table S3. A similar propensity score‐matching (PSM) model was applied to 30‐, 90‐, and 180‐day readmission analyses to calculate readmission rates for matched cohorts. Index hospitalizations with patients alive at discharge were retained for readmission analysis to avoid mortality bias. Using combined data from all years, we used a multivariable logistic regression model described above to obtain predictive margins for the adjusted trends over the years; the year was included as an independent variable. Unadjusted Trend analysis was performed using the Cochran‐Armitage test for binary outcomes (Armitage 1955) and the Jonckheere‐Terpstra test or Cuzick's test for ordered categorical or continuous variables (Cuzick 1985), given the non‐parametric distribution of the study population. The total cost was adjusted for national inflation and merged with the cost‐charge ratio (CCR) NRD files. All analyses were conducted using appropriate stratification, clustering, and weighting, as required by the Healthcare Cost and Utilization Project regulations. Stata v. 18 (StataCorp, College Station, TX) was used for all statistical analyses. We used Biorender for the central illustration.

## 3 Results

### 3.1 Demographic and Baseline Characteristics

Our retrospective analysis was conducted on a cohort of 622,092 hospitalizations with VT in the United States from 2016 to 2020. Of these, 96,755 index VT hospitalizations were included for the 30‐day readmission analysis, and 52,952 index VT hospitalizations were included for the 180‐day readmission analysis.

Of the 622,092 hospitalizations for VT, 575,312 (92.4%) of patients had underlying ICM, while 46,780 (7.5%) had NIDCM. Patients with ICM were significantly older, with a median age of 70 years (IQR: 18 years) compared to 63 years (IQR: 22 years) in the NIDCM group (*p* < 0.001) (Table 1). The majority of patients (70.8% in the ICM group and 65% in the NIDCM group; *p* < 0.001) were male. Most hospitalizations in both groups, ICM versus NIDCM, were non‐elective admissions (96% and 97.4%, respectively; *p* < 0.001). There was a significant difference in the insurance status, with most ICM patients more frequently covered by Medicare (66% vs. 52.7%; *p* < 0.001), whereas Medicaid (19% vs. 8.1%; *p* < 0.001) and private insurance (19.4% vs. 19%; *p* < 0.001) were more common among NIDCM patients. Regarding hospital characteristics, most VT hospitalizations occurred in large hospitals (58.9% in ICM vs. 57.4% in NIDCM; *p* = 0.07) and private, non‐profit hospitals (78.3% vs. 77.2%; *p* = 0.037). The majority of hospitalizations took place in teaching hospitals (74.1% vs. 73.7%; *p* = 0.008). Patients in the NIDCM group were more likely to be treated in large metropolitan hospitals (53.6% vs. 51.7%; *p* < 0.001).

**TABLE 1:** Baseline characteristics and comorbidities comparison of VT in ICM and NIDCM.

Baseline characteristics	VT in ICM	VT in NIDCM	*p*‐value
	*N* = 575,312	*N* = 46,780	
	N (%)	N (%)	
Age (Median + IQR)			
	70 (18)	63 (22)	< 0.001
Indicator of sex			
Male	407,245 (70.8)	30,366 (65)	< 0.001
Female	168,067 (29.2)	16,414 (35)	
Type of admission			
Non‐elective	551,479 (96)	45,525 (97.4)	< 0.001
Elective	23,009 (4)	1221 (2.6)	
Comorbidities			
Diabetes	249,265 (43.3)	14,863 (31.8)	< 0.001
Hyperlipidemia	408,884 (71.1)	18,788 (40.2)	< 0.001
Hypertension	368,718 (64.1)	34,104 (72.9)	< 0.001
Smoker	181,598 (31.6)	11,739 (25.1)	< 0.001
Obesity	113,332 (19.7)	11,512 (24.6)	< 0.001
CKD stage over 3	125,595 (21.8)	9987 (21.3)	0.12
ESRD	32,215 (5.6)	3139 (6.7)	< 0.001
Prior CVA	52,065 (9)	3052 (6.5)	< 0.001
Prior defibrillator	55,996 (9.7)	5347 (11.4)	< 0.001
Prior PPM	24,794 (4.3)	1547 (3.3)	< 0.001
OSA	58,670 (10.2)	5493 (11.7)	< 0.001
Pulmonary disease	127,687 (22.2)	9991 (21.4)	0.007
Pulmonary hypertension	64,989 (11.3)	8384 (17.9)	< 0.001
RVHF	879 (0.2)	192 (0.4)	< 0.001
Hypothyroidism	71,004 (12.3)	4738 (10.1)	< 0.001
Anemia	34,196 (5.9)	3149 (6.7)	< 0.001
Liver disease	13,131 (2.3)	2772 (5.9)	< 0.001
Malnutrition	33,287 (5.8)	4722 (10.1)	< 0.001
Heart failure	406,661 (70.7)	41,462 (88.6)	< 0.001
COVID‐19 infection	3115 (0.5)	528 (1.1)	< 0.001

Abbreviations: CKD, chronic kidney disease; CVA, cerebral vascular accident; ESRD, end stage renal disease; ICM, ischemic cardiomyopathy; NIDCM, non‐ischemic dilated cardiomyopathy; OSA, obstructive sleep apnea; PPM, permanent pacemaker; RVHF, right ventricular heart failure; VT, ventricular tachycardia.

Most patients were admitted on weekdays (73.9% in ICM vs. 74.9% in NIDCM; *p* < 0.001). A higher proportion of NIDCM patients had non‐transfer hospitalizations (91.3% vs. 88.7%; *p* < 0.001). Patients in the lowest household income quartile were more common in the NIDCM group (35.6% vs. 29.4%; *p* < 0.001), while those in the highest income quartile were more common in the ICM group (17.1% vs. 16%; *p* < 0.001). A higher proportion of ICM patients had an extreme risk of mortality (37% vs. 45.6%; *p* < 0.001) and greater severity of illness (33.3% vs. 41%; *p* < 0.001). The prevalence of medical comorbidities significantly differed between the two groups. Patients in the ICM group had a higher prevalence of diabetes (43.3% vs. 31.8%, *p* < 0.001), hyperlipidemia (71.1% vs. 40.2%, *p* < 0.001), and hypertension (64.1% vs. 72.9%, *p* < 0.001). A history of smoking (31.6% vs. 25.1%, *p* < 0.001), chronic kidney disease (CKD) stage > 3 (21.8% vs. 21.3%, *p* = 0.12), and prior cerebrovascular accident (9% vs. 6.5%, *p* < 0.001) was also more frequent in the ICM group. While the presence of a prior defibrillator (9.7% vs. 11.4%, *p* < 0.001) was more common in the NIDCM group, permanent pacemaker implantation (4.3% vs. 3.3%, *p* < 0.001) was more common among ICM patients.

On the other hand, patients with NIDCM had a significantly higher prevalence of heart failure (88.6% vs. 70.7%, *p* < 0.001) and arrhythmias (57.7% vs. 52.7%, *p* < 0.001). End‐stage renal disease (6.7% vs. 5.6%, *p* < 0.001), obstructive sleep apnea (11.7% vs. 10.2%, *p* < 0.001), pulmonary hypertension (17.9% vs. 11.3%, *p* < 0.001), and right ventricular heart failure (0.4% vs. 0.2%, *p* < 0.001) were more common in the NIDCM group. Additional notable differences included a higher prevalence of anemia (6.7% vs. 5.9%, *p* < 0.001), liver disease (5.9% vs. 2.3%, *p* < 0.001), and malnutrition (10.1% vs. 5.8%, *p* < 0.001) in the NIDCM group. Patients with NIDCM also had a higher frequency of COVID‐19 infection (1.1% vs. 0.5%, *p* < 0.001). Baseline characteristics and medical comorbidities are shown in Table 1.

### 3.2 Crude and Propensity Matched in‐Hospital Outcomes of VT in ICM and NIDCM

Patients in the NIDCM group had a higher in‐hospital mortality rate both in crude analysis (10.3% vs. 8.7%, *p* < 0.001) and after propensity matching (10.4% vs. 9.6%, *p* < 0.001). In crude analysis, patients in the ICM group had a higher incidence of Sudden Cardiac Arrest (SCA) (8.1% vs. 6%, *p* < 0.001), Major Adverse Cardiac Event (MACE) (82.1% vs. 53%, *p* < 0.001), need for ablation (0.5% vs. 1%, *p* < 0.001), and cardiogenic shock (12.7% vs. 12%, *p* = 0.01). Additionally, patients in the ICM group had a higher rate of endotracheal intubation (10.5% vs. 14.5%, *p* < 0.001).

Conversely, patients in the NIDCM group had a higher incidence of acute congestive heart failure (CHF) (68.4% vs. 46%, *p* < 0.001), stroke (1.8% vs. 1.1%, *p* < 0.001), Left Ventricular Assist Device (LVAD) utilization (0.3% vs. 0.2%, *p* < 0.001), cardiac transplantation (0.2% vs. 0%, *p* < 0.001), need for ablation (1% vs. 0.5%, *p* < 0.001) and Acute Kidney Injury (AKI) (45.8% vs. 35%, *p* < 0.001).

**FIGURE 2:**
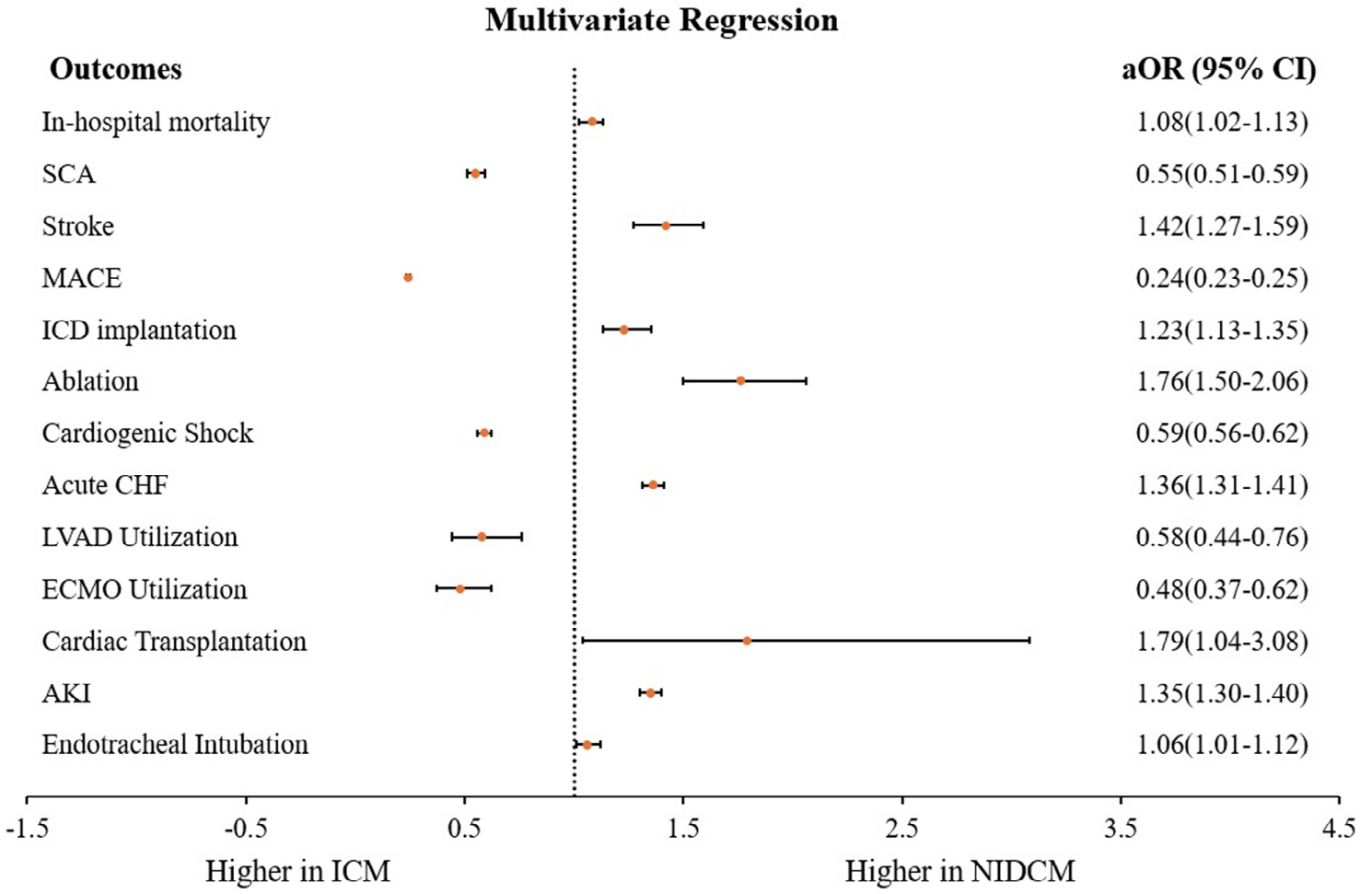
Multivariate regression of VT outcomes in NIDCM vs. ICM patients.

After propensity matching (*N* = 25,919 pairs), all previously observed differences remained statistically significant. The ICM group continued to have a higher incidence of SCA (10.6% vs. 5.9%, *p* < 0.001), MACE (78.9% vs. 52.2%), cardiogenic shock (18.4% vs. 12.1%, *p* < 0.001), and ECMO utilization (0.9% vs. 0.4%, *p* < 0.001). On the other hand, the incidence of endotracheal intubation (14.5% vs. 13.1%, *p* < 0.001) and the need for ablation (1% vs. 0.5%, *p* < 0.001) were higher in the NIDCM group than in the ICM group. The crude and propensity‐matched outcomes are graphically represented in Figure 2 and Figure S3.

Additionally, the patients in the NIDCM group showed a higher incidence of acute congestive heart failure (65% vs. 59%, *p* < 0.001), stroke (1.7% vs. 1.2%, *p* < 0.001), AKI (46.1% vs. 41.7%, *p* < 0.001), and ICD implantation (3.9% vs. 3.5%, *p* = 0.01). The incidence of LVAD utilization was higher in the ICM group (0.5% vs. 0.3%, *p* = 0.001). The incidence of cardiac transplantation (0.2% vs. 0.1%, *p* = 0.29) was not statistically significant after propensity matching. Crude and propensity‐matched outcomes are shown in Table 2.

**TABLE 2:** Crude and propensity matched in‐hospital outcomes of VT in ICM and NIDCM.

Outcomes	Crude outcomes			Propensity match outcomes		
	VT in NIDCM	VT in ICM	*p*‐value	VT in NIDCM	VT in ICM	*p*‐value
	N (%)	N (%)		N (%)	N (%)	
	*N* = 46,780	*N* = 575,312		*N* = 25,919	*N* = 25,919	
Died during hospitalization	4824 (10.3)	50,101 (8.7)	< 0.001	2693 (10.4)	2501 (9.6)	0.005
SCA	2797 (6)	46,469 (8.1)	< 0.001	1525 (5.9)	2758 (10.6)	< 0.001
Stroke	837 (1.8)	6555 (1.1)	< 0.001	453 (1.7)	318 (1.2)	< 0.001
MACE	24,775 (53)	472,176 (82.1)	< 0.001	13,537 (52.2)	20,462 (78.9)	< 0.001
ICD implantation	1804 (3.9)	14,301 (2.5)	< 0.001	1010 (3.9)	900 (3.5)	0.01
Ablation	455 (1)	2653 (0.5)	< 0.001	251 (1.0)	126 (0.5)	< 0.001
Cardiogenic shock	5633 (12)	72,967 (12.7)	0.01	3136 (12.1)	4782 (18.4)	< 0.001
Acute CHF	30,301 (64.8)	264,647 (46)	< 0.001	16,852 (65.0)	15,528 (59.9)	< 0.001
LVAD utilization	163 (0.3)	1337 (0.2)	0.002	85 (0.3)	132 (0.5)	0.001
ECMO utilization	194 (0.4)	2055 (0.4)	0.184	109 (0.4)	239 (0.9)	< 0.001
Cardiac transplantation	78 (0.2)	137 (0)	< 0.001	41 (0.2)	32 (0.1)	0.29
AKI	21,423 (45.8)	201,225 (35)	< 0.001	11,958 (46.1)	10,819 (41.7)	< 0.001
Endotracheal intubation	6791 (14.5)	59,373 (10.3)	< 0.001	3752 (14.5)	3397 (13.1)	< 0.001

Abbreviations: AKI, acute kidney injury; CHF, congestive heart failure; ECMO, extracorporeal membrane oxygenation; ICD, implantable cardiac defibrillator; ICM, ischemic cardiomyopathy; LVAD, left ventricular assist device; MACE, major adverse cardiac event; NIDCM, non‐ischemic dilated cardiomyopathy; SCA, sudden cardiac arrest; VT, ventricular tachycardia.

### 3.3 Outcomes After Multivariate Regression Analysis

On multivariate regression analysis, after adjusting for the confounders, patients in the NIDCM group compared to the ICM group had significantly higher odds of in‐hospital mortality (adjusted odds ratio, aOR: 1.08, 95% CI: 1.02–1.13, *p* < 0.001), stroke (aOR: 1.42, 95% CI: 1.27–1.59, *p* < 0.001), ICD implantation (aOR: 1.23, 95% CI: 1.13–1.35, *p* < 0.001), VT ablation (aOR: 1.76, 95% CI: 1.50–2.06, *p* < 0.001), acute congestive heart failure (aOR: 1.36, 95% CI: 1.31–1.41, *p* < 0.001), cardiac transplantation (aOR: 1.79, 95% CI: 1.04–3.08, *p* = 0.037), acute kidney injury (aOR: 1.35, 95% CI: 1.30–1.40, *p* < 0.001), and endotracheal intubation (aOR: 1.06, 95% CI: 1.01–1.12, *p* = 0.012).

Conversely, the odds of sudden cardiac arrest (aOR: 0.55, 95% CI: 0.51–0.59, *p* < 0.001), major adverse cardiac events (MACE) (aOR: 0.24, 95% CI: 0.23–0.25, *p* < 0.001), cardiogenic shock (aOR: 0.59, 95% CI: 0.56–0.62, *p* < 0.001), LVAD utilization (aOR: 0.58, 95% CI: 0.44–0.76, *p* < 0.001), and ECMO utilization (aOR: 0.48, 95% CI: 0.37–0.62, *p* < 0.001) were lower in the NIDCM group compared to the ICM group. Multivariate regression analysis is shown in Table 3 and represented graphically in Figure 2.

**TABLE 3:** Multivariate regression analysis comparing VT in NIDCM vs ICM.

In‐hospital outcomes	aOR	95% CI	*p*‐value
Died during hospitalization	1.08	1.02–1.13	< 0.001
SCA	0.55	0.51–0.59	< 0.001
Stroke	1.42	1.27–1.59	< 0.001
MACE	0.24	0.23–0.25	< 0.001
ICD implantation	1.23	1.13–1.35	< 0.001
Ablation	1.76	1.50–2.06	< 0.001
Cardiogenic shock	0.59	0.56–0.62	< 0.001
Acute CHF	1.36	1.31–1.41	< 0.001
LVAD utilization	0.58	0.44–0.76	< 0.001
ECMO utilization	0.48	0.37–0.62	< 0.001
Cardiac transplantation	1.79	1.04–3.08	0.037
AKI	1.35	1.30–1.40	< 0.001
Endotracheal intubation	1.06	1.01–1.12	0.012

Abbreviations: AKI, acute kidney injury; CHF, congestive heart failure; ECMO, extracorporeal membrane oxygenation; ICD, implantable cardiac defibrillator; ICD, implantable cardiac defibrillator; ICM, ischemic cardiomyopathy; LVAD, left ventricular assist device; MACE, major adverse cardiac event; NIDCM, non‐ischemic dilated cardiomyopathy; SCA, sudden cardiac arrest; VT, ventricular tachycardia.

### 3.4 Readmission Rates on Propensity Matched Cohort of VT in ICM and NIDCM

On a propensity‐matched cohort, the NIDCM cohort has a higher rate of all‐cause readmission at 30‐day (10.4% vs. 8.7%, p: 0.019), 90‐day (22.7% vs. 20%, p: 0.483), and 180‐day (30.7% vs. 27.3%, p: 0.001) intervals. Readmission rates on propensity‐matched cohorts are shown in Table 4. Heart failure‐related readmission rates are shown in Table 5.

**TABLE 4:** Readmission rates on propensity matched cohort.

Readmission rates on propensity matched cohort			
30 day Readmissions	VT in ICM	VT in NIDCM	*p*‐value
	*N* = 25,919	*N* = 25,919	
	N (%)	N (%)	
Readmits	2250 (8.7)	2689 (10.4)	< 0.001
90 day Readmissions	VT in ICM	VT in NIDCM	*p*‐value
	*N* = 21,054	*N* = 21,054	
	N (%)	N (%)	
Readmits	4312 (20.5)	4771 (22.7)	< 0.001
180 day Readmissions	VT in ICM	VT in NIDCM	*p*‐value
	*N* = 14,414	*N* = 14,414	
	N (%)	N (%)	
Readmits	3931 (27.3)	4422 (30.7)	< 0.001

Abbreviations: ICM, ischemic cardiomyopathy; NIDCM, non‐ischemic dilated cardiomyopathy; VT, ventricular tachycardia.

**TABLE 5:** Heart failure‐related readmission rates due to HF on propensity matched cohort.

Readmission rates on propensity matched cohort			
30 day Readmissions	VT in ICM	VT in NIDCM	*p*‐value
	*N* = 16,852	*N* = 16,852	
	N (%)	N (%)	
Readmits	1540 (9.1)	1852 (11)	< 0.001
90 day Readmissions	VT in ICM	VT in NIDCM	*p*‐value
	*N* = 13,699	*N* = 13,699	
	N (%)	N (%)	
Readmits	2847 (20.8)	3270 (23.9)	< 0.001
180 day Readmissions	VT in ICM	VT in NIDCM	*p*‐value
	*N* = 9456	*N* = 9456	
	N (%)	N (%)	
Readmits	2770 (29.3)	3026 (32)	< 0.001

Abbreviations: HF, heart failure; ICM, ischemic cardiomyopathy; NIDCM, non‐ischemic dilated cardiomyopathy; VT, ventricular tachycardia.

### 3.5 Yearly Trend of Mortality, SCA, ICD Implantation, and Ablation of VT in ICM and NIDCM

From 2016 to 2020, there was a significant decrease in mortality rate in the ICM cohort (8.5% in 2016 to 8.2% in 2020, p‐trend: 0.006), whereas there was no significant change in the NIDCM cohort (12.6% in 2016 to 12.6% in 2020, p‐trend: 0.515). Additionally, there was a significant decrease in the incidence of SCA in the ICM cohort (8.0% in 2016 to 7.6% in 2020, p‐trend: < 0.001), while the NIDCM cohort did not show a significant change (6.6% in 2016 to 6.1% in 2020, p‐trend: > 0.067). The rate of ICD implantation significantly decreased in the ICM cohort (1.9% in 2016 to 1.6% in 2020, p‐trend: 0.02), while there was no significant change in the NIDCM cohort (2.8% in 2016 to 2.2% in 2020, p‐trend: 0.067). The incidence of VT ablation significantly increased in the ICM cohort (0.2% in 2016 to 0.4% in 2020, p‐trend: 0.024), whereas there was no significant change in the NIDCM cohort (0.9% in 2016 to 1.2% in 2020, p‐trend: 0.085) (Table 6).

**TABLE 6:** Yearly trend of mortality, SCA, ICD implantation, and ablation in VT in ICM and NIDCM.

Year	VT in ICM				VT in NIDCM			
	Mortality (%)	SCA (%)	ICD implantatio*n* (%)	Ablation (%)	Mortality (%)	SCA (%)	ICD implantatio*n* (%)	Ablation (%)
2016	8.5	8.0	1.9	0.2	12.6	6.6	2.8	0.9
2017	8.5	8.4	1.6	0.3	12.7	6.9	2.7	0.8
2018	8.2	7.5	1.6	0.3	11.7	5.4	2.8	0.9
2019	8.1	7.2	1.6	0.3	12.8	5.5	2.6	0.7
2020	8.2	7.6	1.6	0.4	12.6	6.1	2.2	1.2
P‐trend	0.006	< 0.001	0.02	0.024	0.515	0.067	0.067	0.085

Abbreviations: ICD, implantable cardiac defibrillator; ICM, ischemic cardiomyopathy; NIDCM, non‐ischemic dilated cardiomyopathy; SCA, sudden cardiac arrest; VT, ventricular tachycardia.

### 3.6 Predictors of Readmissions

In multivariable regression analyses, VT in non‐ischemic dilated cardiomyopathy (NIDCM) was independently associated with higher odds of readmission at 30 days (aOR 1.23, 95% CI 1.17–1.29), 90 days (aOR 1.17, 95% CI 1.13–1.21), and 180 days (aOR 1.19, 95% CI 1.14–1.24) (all *p* < 0.001). Across all time points, female sex, diabetes, advanced chronic kidney disease, end‐stage renal disease, heart failure, prior cerebrovascular accident, implanted cardiac devices, anemia, pulmonary disease, pulmonary hypertension, and hypothyroidism were consistently associated with increased readmission risk. In contrast, hyperlipidemia and obesity were associated with lower odds. COVID‐19 infection was associated with reduced readmissions at 30, 90, and 180 days. Compared with Medicare, private insurance, self‐pay, and no‐charge status were associated with lower readmission risk, while Medicaid was associated with higher odds at 30 and 180 days. Metropolitan teaching hospitals were associated with modestly lower readmissions, whereas private investor‐owned hospitals and larger bed size were associated with higher odds of readmission at longer follow‐up intervals (Tables 7, 8, 9).

**TABLE 7:** Multivariate regression analysis for predictors of 30‐day readmission.

Predictors of Readmission—30d	aOR	95% CI	*p*‐value
Comorbidites			
VT in NIDCM	1.23	1.17–1.29	< 0.001
Female gender	1.05	1.02–1.09	< 0.001
Diabetes	1.14	1.10–1.17	< 0.001
Hyperlipidemia	0.93	0.91–0.96	< 0.001
Hypertension	1.08	1.04–1.13	< 0.001
CKD Stage ≥ 3	1.26	1.21–1.30	< 0.001
ESRD	1.56	1.48–1.64	< 0.001
Arrhythmias	1.05	1.02–1.08	< 0.001
Obesity	0.92	0.89–0.96	< 0.001
Prior CVA	1.13	1.08–1.19	< 0.001
Prior PPM	1.18	1.11–1.26	< 0.001
Prior defibrillator	1.42	1.36–1.48	< 0.001
OSA	1.05	1.01–1.10	0.043
Pulmonary disease	1.34	1.30–1.39	< 0.001
Pulmonary hypertension	1.12	1.07–1.16	< 0.001
Hypothyroidism	1.09	1.05–1.14	< 0.001
Anemia	1.22	1.16–1.29	< 0.001
Liver disease	1.19	1.10–1.29	< 0.001
Heart failure	1.47	1.40–1.54	< 0.001
Covid infection	0.73	0.60–0.90	< 0.001
Insurance Type^a^			
Medicaid	1.05	1.01–1.12	0.048
Private insurance	0.65	0.62–0.68	< 0.001
Self‐Pay	0.68	0.62–0.75	< 0.001
No charge	0.75	0.59–0.95	0.017
Other	0.73	0.66–0.80	< 0.001
Hospital characteristics: Hospital urban–rural designation^b^			
Metropolitan teaching	0.92	0.88–0.95	< 0.001
Hospital characteristics: Control/Ownership of hospital^c^			
Private, invest‐own	1.11	1.04–1.19	< 0.001
A combined record involving rehabilitation transfer			
Rehabilitation transfer	0.29	0.24–0.35	< 0.001

Abbreviations: aOR, adjusted odds ratio; CI, confidence interval; CKD, chronic kidney disease; CVA, cerebral vascular accident; ESRD, end stage renal disease; OSA, obstructive sleep apnea; PPM, permanent pacemaker; VT in NIDCM, ventricular tachycardia in non‐ischemic dilated cardiomyopathy.

^a^ Compared to Medicare.

^b^ Compared to large metropolitan areas with at least 1 million residents.

^c^ Compared to Government/Non‐Federal Hospitals.

**TABLE 8:** Multivariate regression analysis for predictors of 90‐day readmission.

Predictors of Readmission—90d	aOR	95% CI	*p*‐value
Comorbidites			
VT in NIDCM	1.17	1.13–1.21	< 0.001
Female gender	1.09	1.07–1.12	< 0.001
Diabetes	1.20	1.18–1.23	< 0.001
Hyperlipidemia	0.96	0.93–0.98	< 0.001
Hypertension	1.07	1.04–1.11	< 0.001
Obesity	0.95	0.93–0.98	< 0.001
CKD stage over 3	1.28	1.25–1.32	< 0.001
ESRD	1.61	1.54–1.68	< 0.001
Prior CVA	1.16	1.12–1.20	< 0.001
Prior PPM	1.15	1.09–1.21	< 0.001
Arrhythmias	1.09	1.06–1.11	< 0.001
Prior PPM	1.15	1.09–1.21	< 0.001
Prior defibrillator	1.36	1.32–1.41	< 0.001
OSA	1.14	1.10–1.18	< 0.001
Pulmonary disease	1.35	1.31–1.38	< 0.001
Pulmonary hypertension	1.14	1.10–1.18	< 0.001
Hypothyroidism	1.08	1.05–1.12	< 0.001
Anemia	1.30	1.24–1.35	< 0.001
Liver disease	1.14	1.07–1.22	< 0.001
Malnutrition	1.10	1.05–1.15	< 0.001
Heart failure	1.52	1.47–1.58	< 0.001
Covid infection	0.63	0.53–0.74	< 0.001
Insurance type^a^			
Private insurance	0.65	0.62–0.67	< 0.001
Self‐pay	0.64	0.60–0.69	< 0.001
No charge	0.70	0.57–0.87	< 0.001
Other	0.69	0.64–0.73	< 0.001
Hospital characteristics: Hospital bed size^b^			
Large	1.05	1.01–1.09	< 0.001
Hospital characteristics: Hospital urban–rural designation^c^			
Metropolitan teaching	0.95	0.92–0.98	< 0.001
Not metropolitan	0.90	0.85–0.95	< 0.001
Hospital characteristics: Control/Ownership of hospital^d^			
Private, invest‐own	1.08	1.02–1.14	< 0.001
A combined record involving rehabilitation transfer			
Rehabilitation transfer	0.86	0.78–0.94	< 0.001

Abbreviations: aOR, adjusted odds ratio; CI, confidence interval; CKD, chronic kidney disease; CVA, cerebral vascular accident; ESRD, end stage renal disease; OSA, obstructive sleep apnea; PPM, permanent pacemaker; VT in NIDCM, ventricular tachycardia in non‐ischemic dilated cardiomyopathy.

^a^ Compared to Medicare.

^b^ Compared to the hospitals with small bed size.

^c^ Compared to large metropolitan areas with at least 1 million residents.

^d^ Compared to Government/Non‐Federal Hospitals.

**TABLE 9:** Multivariate regression analysis for predictors of 180‐day readmission.

Predictors of Readmission—180d	aOR	95% CI	*p*‐value
Comorbidites			
VT in NIDCM	1.19	1.14–1.24	< 0.001
Female gender	1.10	1.07–1.13	< 0.001
Diabetes	1.23	1.20–1.26	< 0.001
Hyperlipidemia	0.96	0.94–0.99	< 0.001
Hypertension	1.10	1.06–1.13	< 0.001
Obesity	0.95	0.92–0.98	< 0.001
CKD stage over 3	1.29	1.25–1.33	< 0.001
ESRD	1.63	1.56–1.71	< 0.001
Arrhythmias	1.05	1.02–1.07	< 0.001
Prior CVA	1.16	1.11–1.21	< 0.001
Prior PPM	1.24	1.17–1.31	< 0.001
Prior defibrillator	1.42	1.37–1.47	< 0.001
OSA	1.16	1.11–1.21	< 0.001
Pulmonary disease	1.35	1.31–1.39	< 0.001
Pulmonary hypertension	1.14	1.10–1.18	< 0.001
Hypothyroidism	1.09	1.05–1.13	< 0.001
Anemia	1.27	1.21–1.33	< 0.001
Liver disease	1.08	1.01–1.16	0.029
Heart failure	1.48	1.43–1.54	< 0.001
Malnutrition	1.07	1.02–1.12	< 0.001
Smoker	1.03	1.01–1.06	0.017
Covid infection	0.61	0.48–0.78	< 0.001
Insurance type^a^			
Medicaid	1.06	1.01–1.11	0.013
Private insurance	0.63	0.60–0.66	< 0.001
Self‐pay	0.67	0.62–0.73	< 0.001
No charge	0.71	0.58–0.88	< 0.001
Other	0.64	0.59–0.69	< 0.001
Hospital characteristics: Hospital bed size^b^			
Large	1.05	1.01–1.10	0.014
Hospital characteristics: Hospital urban–rural designation^c^			
Metropolitan teaching	0.95	0.92–0.98	< 0.001
Not metropolitan	0.90	0.85–0.96	< 0.001
Hospital characteristics: Control/Ownership of hospital^d^			
Private, invest‐own	1.06	1.01–1.12	0.049

Abbreviations: aOR, adjusted odds ratio; CABG, coronary artery bypass graft; CI, confidence interval; CKD, chronic kidney disease; CVA, cerebral vascular accident; ESRD, end stage renal disease; MI, myocardial infarction; OSA, obstructive sleep apnea; PCI, percutaneous coronary intervention; PPM, permanent pacemaker; VT in NIDCM, ventricular tachycardia in non‐ischemic dilated cardiomyopathy.

^a^ Compared to Medicare.

^b^ Compared to the hospitals with small bed size.

^c^ Compared to large metropolitan areas with at least 1 million residents.

^d^ Compared to Government/Non‐Federal Hospitals.

## 4 Discussion

**FIGURE 3:**
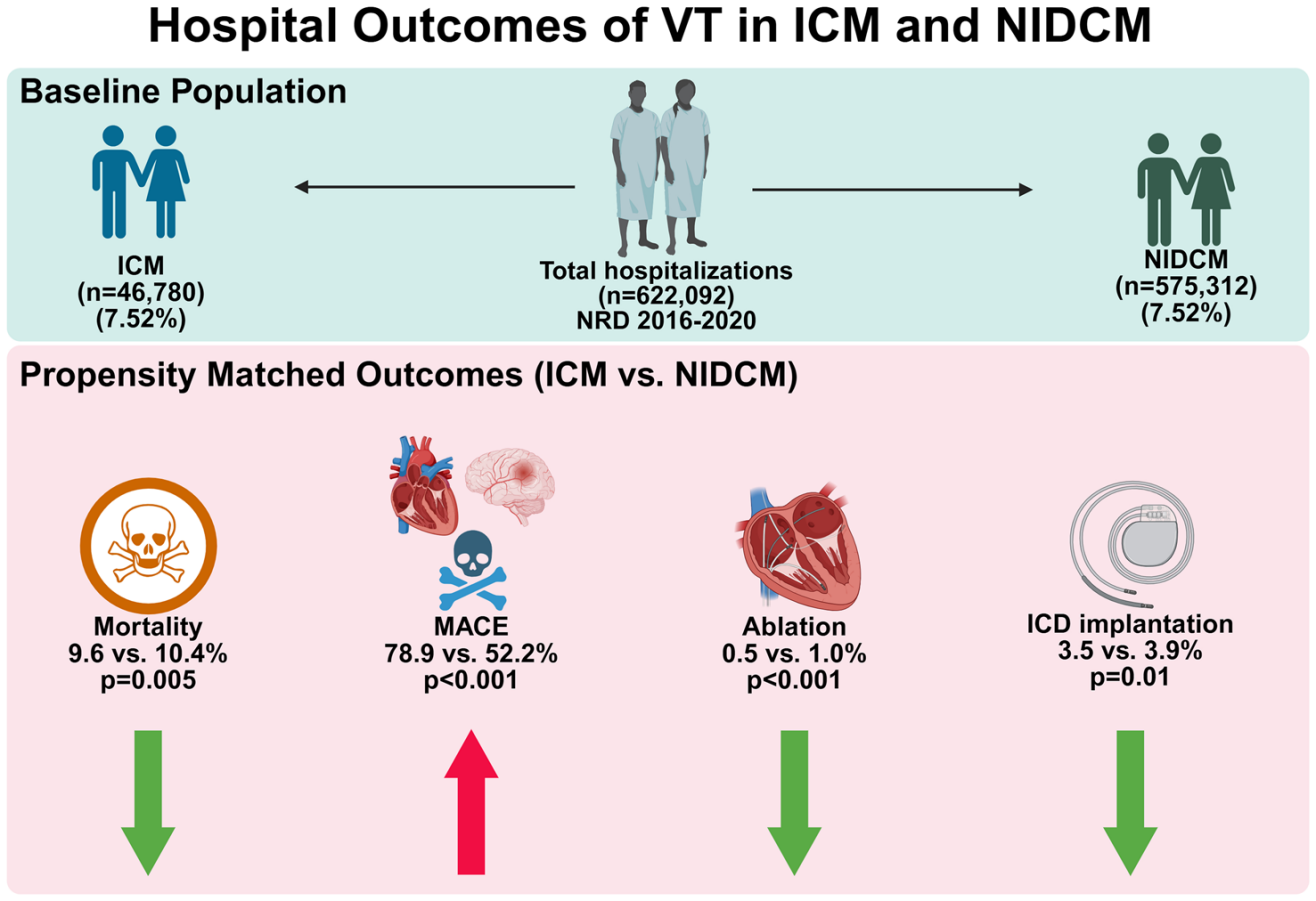
Central illustration summarizing the baseline population and propensity‐matched in‐hospital outcomes of ventricular tachycardia (VT) hospitalizations in patients with ischemic cardiomyopathy (ICM) and non‐ischemic dilated cardiomyopathy (NIDCM).

Our analysis of 622,092 index hospitalizations for ventricular tachycardia (VT) revealed distinct differences between patients with ischemic cardiomyopathy (ICM) and those with non‐ischemic dilated cardiomyopathy (NIDCM). Patients with ICM were generally older and had a higher burden of comorbidities. In contrast, despite having lower rates of sudden cardiac arrest (SCA) and major adverse cardiovascular events (MACE), patients with NIDCM experienced higher in‐hospital mortality, increased rates of stroke, more heart failure‐related admissions, and greater readmission rates. Additionally, ICD implantation and ablation procedures were more frequently performed in the NIDCM group. A central illustration summarizing the findings is shown in Figure 3.

Our finding of higher mortality in the NIDCM group contrasts with prior literature, including European registry data, where patients with ICM demonstrated higher mortality (Tymińska et al. 2022). This trend persisted even among patients who received cardiac resynchronization therapy, as noted by (McLeod et al. 2011). Several factors may explain this discrepancy. First, our cohort may have included a sicker NIDCM population, such as those with myocarditis, fulminant cardiomyopathies, or toxin‐related myopathies, which are known to be associated with higher short‐term mortality (Ammirati et al. 2020; Caforio et al. 2013). The inclusion period of 2016–2020 also coincides with the COVID‐19 pandemic, which may have increased the prevalence of virus‐related myocarditis in the NIDCM group, particularly in 2020 (Siripanthong et al. 2020). The relatively small proportion of NIDCM hospitalizations (7.5%) may also reflect the specificity of our administrative case definition. Because NIDCM required a positive I42.0 code and exclusion of coded obstructive CAD, prior PCI, and CABG, undercoding of coronary disease could result in some ischemic patients being classified as NIDCM, whereas patients with NIDCM documented only under more nonspecific cardiomyopathy or heart failure codes would not be captured. The former would be expected to attenuate differences between groups by introducing patients with ischemic disease into the NIDCM cohort, while the latter would reduce the representativeness of the NIDCM cohort. The magnitude of this potential misclassification cannot be estimated using the NRD.

Conversely, the lower mortality observed in ICM may reflect recent advances in ischemic heart disease management. Prior retrospective studies have indicated that in patients with ST‐elevation myocardial infarction, incomplete revascularization was associated with a higher risk of future arrhythmias (Thomsen et al. 2023). In addition, the COMPLETE trial demonstrated that complete revascularization in patients with multivessel coronary artery disease significantly reduced cardiovascular events (Mehta et al. 2019). The role of coronary revascularization in reducing ventricular arrhythmias (VAs) in stable CAD remains uncertain. Major trials such as REVIVED‐BCIS2 and VANISH found no reduction in VA incidence with revascularization, likely due to fixed scar substrates and advanced disease (Perera et al. 2023; Elsokkari et al. 2018). These studies were not powered for arrhythmic outcomes and were limited by heterogeneity in patient populations and VA definitions. However, observational data suggest a potential benefit in anatomically selected patients. Specifically, revascularization of infarct‐related chronic total occlusions (CTOs) was associated with lower VA rates and ICD therapies, highlighting a possible role in modifying arrhythmogenic substrate in select cases (Junarta et al. 2025). Mortality in ICM may also be impacted by earlier recognition of hemodynamic compromise and the availability of structured shock management protocols, leading to prompt initiation of circulatory support such as ECMO or Impella. These findings align with the FRENSHOCK registry, where ICM patients more frequently received ECMO in the setting of acute myocardial infarction‐related shock, likely due to the reversible nature of their presentations (Cherbi et al. 2023).

Our analysis demonstrated a higher prevalence of ICD implantation among patients with NIDCM compared to those with ischemic cardiomyopathy (ICM) (11.4% vs. 9.7%). Notably, despite a higher baseline ICD use in the NIDCM cohort, the incidence of ICD implantation during index hospitalization was also elevated (3.9% vs. 3.5%). This association remained significant after multivariable adjustment, with NIDCM patients exhibiting increased odds of ICD implantation (aOR 1.23; 95% CI: 1.13–1.35; *p* < 0.001). The greater use of ICD interventions in NIDCM likely reflects the distinct arrhythmogenic substrate in this population. VT in NIDCM is more frequently associated with diffuse fibrosis and non‐transmural scarring, often involving epicardial or intramural regions. In contrast, VT in ICM typically arises from well‐demarcated endocardial scars related to prior infarction and is more amenable to standard endocardial ablation. These pathophysiological differences can complicate both mapping and ablation in NIDCM (Shirai et al. 2019).

Although acute success and VT termination rates are similar between ICM and NIDCM, long‐term outcomes in NIDCM remain poorer. Previous studies have reported lower rates of complete procedural success—defined by non‐inducibility at the end of the procedure—and reduced one‐year VT‐free survival in NIDCM patients (Dinov et al. 2014). Interestingly, while the adoption of more advanced ablation strategies—such as combined endocardial–epicardial (endo‐epi) approaches—has begun to increase in recent years, particularly during the latter part of our study period (2016–2020) (Bhaskaran et al. 2020), it is likely that the full implementation and impact of these techniques have not yet been realized. As such, improvements in VT outcomes and mortality associated with these strategies may not be fully captured within the timeframe of our analysis. Additionally, this trend may likely reflect a selection bias toward sicker patients with more complex substrates undergoing these procedures, as well as the progressive nature of NIDCM itself (Gulati et al. 2013).

Consistent with these findings, our study also observed higher 30‐ and 180‐day readmission rates among NIDCM patients. These trends may partly explain the lower utilization of catheter ablation during index hospitalization in this group, despite their higher arrhythmic burden and increased rates of ICD interventions. Although obesity was more prevalent in NIDCM than ICM, it was included as a covariate in the multivariable readmission models and was independently associated with lower odds of readmission, consistent with the obesity paradox described in heart failure populations. Thus, obesity may contribute to the observed readmission pattern but is unlikely to fully account for the higher readmission odds associated with NIDCM.

### 4.1 Limitations

This study has several important limitations. First, given its retrospective design, it is subject to inherent selection bias. Second, patient identification relied on ICD‐10 codes, which are susceptible to misclassification and coding errors. Third, the Nationwide Readmission Database (NRD) does not include laboratory values, hemodynamic parameters, or echocardiographic data—key clinical variables that may influence outcomes and risk stratification. Lastly, the database lacks temporal resolution, limiting our ability to determine the exact sequence of interventions, complications, and clinical events during hospitalization. In addition, the combined I47.0/I47.2 definition of VT could not be validated against chart or electrocardiographic data, and we were unable to perform a sensitivity analysis restricted to I47.2. The administrative definition of NIDCM may also result in bidirectional misclassification, as undercoding of CAD may include occult ischemic disease in the NIDCM group, while requiring an I42.0 code may exclude patients with NIDCM documented using less specific cardiomyopathy or heart failure codes. These limitations may attenuate or otherwise distort between‐group differences, and their magnitude cannot be quantified in the NRD.

Taken together, these limitations underscore the need for cautious interpretation of our findings and highlight the importance of prospective studies to validate these observations further.

## 5 Conclusion

In this national cohort of patients hospitalized with VT, those with NIDCM had higher illness severity, in‐hospital mortality, and readmission rates despite lower rates of cardiogenic shock and ECMO use compared to ICM. NICM was also associated with greater use of VT ablation and ICD insertion. In contrast, ICM patients had higher rates of SCA and MACE but experienced lower overall mortality, potentially reflecting improved access to revascularization and established acute care pathways.

## Author Contributions

Medhat Chowdhury and Sanchit Duhan: writing – original draft, review and editing, visualization, software. Rasheed Bahar: formal analysis, data curation, writing – review and editing. Mohamad Hasan Jawadi and Bijeta Keisham: writing – original draft. Harini Lakshman, Marc Zughaib, Yasar Sattar, Christopher Bradley, and Marcel Zughaib: supervision, validation, writing – review and editing.

## Conflicts of Interest

The authors declare no conflicts of interest.

## Supporting information


**Table S1:** ICD‐10 codes for cohort identification and comorbidities.
**Table S2:** Definitions of outcomes of interest.
**Table S3:** Variables used for Propensity Score Matching.
**Table S4:** Variables used in multivariate logistics regression.
**Figure S1:** Histogram Showing Distribution of Study Population by Age and mortality in the Index hospitalization.
**Figure S2:** Balance Plot for Propensity Matching in VT Patients comparing ICM and NICM.
**Figure S3:** Bar Graph Showing Crude Outcomes.

## Data Availability

The data that support the findings of this study are available in Nationwide readmission database at https://hcup‐us.ahrq.gov/nrdoverview.jsp. These data were derived from the following resources available in the public domain: Online, https://hcup‐us.ahrq.gov/nrdoverview.jsp.
