# Supplementary material for: Trends and In‐Hospital Outcomes of Ventricular Tachycardia in Ischemic Versus Non‐Ischemic Dilated Cardiomyopathy—An Insight From the Nationwide Readmissions Database (2016–2020)
Source: Ann Noninvasive Electrocardiol. 2026 Aug 31;31(5):e70232. doi: 10.1111/anec.70232 (PMC13527730; doi:10.1111/anec.70232)
Supplement: Supplementary file 1 [file ANEC-31-e70232-s001.docx]

**`Supplementary Appendix**

**Title: Trends and In-hospital Outcomes Of Ventricular Tachycardia in Ischemic Versus Non-ischemic Dilated Cardiomyopathy – An Insight from the Nationwide Readmissions Database (2016 – 2020)**

| Description | Content |  |
| --- | --- | --- |
| ICD-10 codes for cohort identification and comorbidities | Table S1 |  |
| Definitions of outcomes of interest | Table S2 |  |
| Variables used for Propensity Score Matching | Table S3 |  |
| Variables used in multivariate logistics regression | Table S4 |  |
| Histogram Showing Distribution of Study Population by Age and mortality in the Index hospitalization | Figure S1 |  |
| Balance Plot for Propensity Matching in VT Patients comparing ICM and NICM | Figure S2 |  |
| Bar Graph Showing Crude Outcomes | Figure S3 |  |

Table S1. ICD-10 codes for cohort identification and comorbidities

| **Study variables** | **ICD-10 Code** |
| --- | --- |
| VT | I47.2, I47.02 |
| ICM | I25.5 |
| Dilated Cardiomyopathy | I42.0 |
| History of Prior CABG | Z951 |
| History of prior PCI | Z955 |
| Obstructive CAD | I2510, I2511, I256, I2581, I2582 |
| Stroke | I63.XX |
| MCS | 5A02110, 5A02210, 5A0211D, 02HA3RZ, 5A02116, 5A0221D, 5A1522F 5A1522G, 5A1522H, 5A15A2F, 5A15A2G, 5A15A2H, |
| AKI | N170, N171, N172, N178, N179, N1 |
| Pericardiac Complications | Cardiac Tamponade: I314, Hemopericardium: I312, Pericarditis: I30, I31, Pericardial Drainage: 0W9D30Z, 0W9D3ZX, 0W9D3ZZ |
| Myocardial Infarction | I2101, I2102, I2109, I2111, I2119, I2121, I2129, I213, I214 |
| Sudden Cardiac Arrest | I46, I97, 029, 004, 007 |
| ICD | 02H43KZ, 02H63KZ, 02H64KZ, 02H70KZ, 02H73KZ, 02H74KZ, 02HK3KZ, 02HK4KZ, 02HL0KZ, 02HL3KZ, 02HL4KZ, 0JH608Z, 0JH609Z, 0JH60FZ, 0JH638Z, 0JH639Z, 0JH63FZ, 0JH808Z, 0JH809Z, 0JH838Z, 0JH839Z |
| VT Ablation | 02583ZZ, 02573ZZ, 02553ZZ, 02563ZZ |
| Cardiogenic shock | R570, T8111XA, T8111XS |
| Acute CHF | I5021, I5023, I5031, I5033, I5041, I5043 |
| LVAD Utilization | 02HA0QZ, 02HA3QZ, 02HA4QZ, 02PA0RZ, 02WA0QZ |
| ECMO Utilization | 5A1522F, 5A1522G, 5A1522H, 5A1522J, 5A15223 |
| Cardiac Transplantation | 02YA0Z0, 02YA0Z1, 02YA0Z2 |
| Intubation | 0BH17EZ, 0BH18EZ |
| Obesity | E66, Z683, Z684, R939, Z6854, 09921 |
| Hypertension | I10, I1150, I1151, I1152, I1158, I1159 |
| Diabetes Mellitus | E08-E13 family |
| Alcohol use | F101, F1010, F1011, F1012, F10120, F10121, F10129, F1014, F1015, F10150, F10151, F10159, F1018, F10180, F10181, F10182, F10188, F1019, F102, F1020, F1021, F1022, F10220, F10221, F10229, F1023, F10230, F10231, F10232, F10239, F1024, F1025, F10250, F10251, F10259, F1026, F1027, F1028, F10280, F10281, F10282, F10288, F1029, F10920, F10921, F10929, F1094, F1095, F10950, F10951, F10959, F1096, F1097, F1098, F10980, F10981, F10982, F10988, F1099 |
| Atrial fibrillation/flutter | I48 family |
| Heart failure | I50, I501, I502, I5020, I5021, I5022, I5023, I503, I5030, I5031, I5032, I5033, I504, I5040, I5041, I5042, I5043, I508, I5081, I50810, I50811, I50812, I50813, I50814, I5082, I5083, I5084, I5089, I509 |
| Chronic obstructive pulmonary disease | J449 |
| Smoker | F17, Z87.891 |
| Chronic kidney disease stage ≥ 3 | N183, N184, N185, E082, E132, I12, I13 |
| ESRD | N186, Z992, Z4931, Z4901 |
| Anemia | D50, D51, D52, D53, D55, D56, D57, D58, D59, D60, D61, D62, D63, D64, D46.0, D46.1, D46.2, D46.4, O99.0 |
| Obstructive sleep apnea | G47.33 |
| History of stroke | I69.3, Z86.73 |
| History of prior ICD | Z95.810 |
| Peripheral vascular disease | E08.5, E09.5, E10.5, E11.5, E13.5, I73, T82.856, Z98.62, Z95.820, I25.2, I25.83 |

**All codes are available at “**[**https://www.icd10data.com/**](https://www.icd10data.com/)**”** AMI Acute Myocardial Infarction, pLVAD: Percutaneous Left Ventricular Assist Device, VA-ECMO: Veno-arterial Extracorporeal Membrane Oxygenation, IABP: Intra-aortic Baloon Pump, CABG: Coronary Artery Bypass Grafting, PCI: Percutaneous Coronary Intervention, PCA: Percutaneous Coronary Angiogram MCS: Mechanical Circulatory Support, ESRD: End Stage Renal Disease, BMI: Body Mass Index, CHF: Congestive heart failure, ICD: Implantable cardioverter defibrillator

**Table S2.** Definition of Major Outcomes

| Outcomes | Definition |
| --- | --- |
| Mortality | All causes of death, including cardiovascular causes such as sudden cardiac death, death due to acute myocardial infarction, heart failure or cardiogenic shock, and non-cardiovascular causes. |
| VT | Patients admitted with ventricular tachycardia upon arrival or patients who subsequently developed ventricular tachycardia during the index hospitalization |
| ICM | Patients with a principle diagnosis of ischemic cardiomyopathy by left ventricular dysfunction (typically LVEF ≤40%) due to significant coronary artery disease, prior myocardial infarction, or previous coronary revascularization (PCI/CABG), leading to chronic heart failure. |
| NICM | Non-ischemic cardiomyopathy due to dilated cardiomyopathy |
| MACE | Major Adverse Cardiovascular and Cerebrovascular Event; MACCE includes in-hospital mortality, myocardial infarction, cardiogenic shock and stroke. |
| Acute CHF | Heart Failure in the setting of ventricular tachycardia |
| AKI | Any acute kidney injury in VT patients during the hospital stay |
| Cardiogenic Shock | Shock resulting from primary failure of the heart in its pumping function, such as in myocardial infarction, severe ischemic cardiomyopathy, mechanical obstruction, or compression of the heart in the setting of VT |
| Mechanical Circulatory Support: | Any type of circulatory support with an intra-aortic balloon pump, impeller pump, ECMO, or external heart assist system utilization during the index hospitalization |
| LOS | The entire length of stay the patient spent in the hospital during the admission. |
| Cost of hospitalization | The total adjusted amount that the hospital got reimbursed for providing their services for the duration of hospitalization |

LOS: Length of Stay, VT: Ventricular tachycardia, ICM: ischemic cardiomyopathy; NICM: Non-ischemic cardiomyopathy; AKI: Acute Kidney Injury,

**Table S3.** Variables used for Propensity Score Matching

Variables included in Propensity Matching

Age

Gender

Rehab Transfer

Resident

Elective/Non-elective admissions

Hospital Bed Size

Hospital Location & Teaching Status

Hospital Region

Weekend Admission

Payer

Pulmonary hypertension

Hypertension

Chronic Pulmonary Disease

OSA

Diabetes

Hyperlipidemia

CKD Stage >3

ESRD

Obesity

Smoking

Prior CVA

Prior MI, PCI, and CABG

Hypothyroidism

Chronic HF

Liver disease

Arrhythmias

Pneumonia

COVID-19

Abbreviations: OSA; Obstructive sleep apnea, CKD; Chronic kidney disease, ESRD; End-Stage renal disease, CAD; Coronary artery disease, HF; Heart failure, CVA; Cerebral vascular accident, MI; Myocardial infarction, PCI; Percutaneous coronary intervention, CABG; Coronary artery bypass grafting

**Table S4.** Variables used for Multivariate Regression

Variables included Multivariate Regression

Age

Gender

Rehab Transfer

Resident

Elective/Non-elective admissions

Hospital Bed Size

Hospital Location & Teaching Status

Hospital Region

Weekend Admission

Payer

Pulmonary hypertension

Hypertension

Chronic Pulmonary Disease

OSA

Diabetes

Hyperlipidemia

CKD Stage >3

ESRD

Obesity

Smoking

Prior CVA

Prior MI, PCI, and CABG

Hypothyroidism

Chronic HF

Liver disease

Arrhythmias

Pneumonia

COVID-19

Abbreviations: OSA; Obstructive sleep apnea, CKD; Chronic kidney disease, ESRD; End-Stage renal disease, CAD; Coronary artery disease, HF; Heart failure, CVA; Cerebral vascular accident, MI; Myocardial infarction, PCI; Percutaneous coronary intervention, CABG; Coronary artery bypass grafting

**Figure S1**: Histogram Showing Distribution of Study Population by Age and mortality in the Index hospitalization

**Figure S2**: Balance Plot for Propensity Matching in AMI-CS Patients Requiring Mechanical Circulatory Support

**Figure S3**: Bar graph Showing Crude and Propensity matched outcomes of ICM vs NIDCM in patients with ventricular tachycardia
